# Legacy Chemical Pollutants in House Dust of Homes of Pregnant African Americans in Atlanta

**DOI:** 10.3390/toxics10120755

**Published:** 2022-12-03

**Authors:** Kathryn J. Barr, Cierra L. Johnson, Jordan Cohen, Priya D’Souza, Estefani Ignacio Gallegos, Chia-Chen Tsai, Anne L. Dunlop, Elizabeth J. Corwin, Dana Boyd Barr, P. Barry Ryan, Parinya Panuwet

**Affiliations:** 1 Department of Environmental Sciences, College of Arts and Sciences, Emory University, Atlanta, GA 30322, USA; 2 Laboratory of Exposure Assessment and Development for Environmental Research, Gangarosa Department of Environmental Health, Rollins School of Public Health, Emory University, Atlanta, GA 30322, USA; 3 Division of Preventive Medicine, School of Medicine, Emory University, Atlanta, GA 30322, USA; 4 School of Nursing, Columbia University, New York, NY 10032, USA

**Keywords:** dust, persistent organic pollutants, Atlanta, African American, pregnant

## Abstract

We developed and applied a method for measuring selected persistent organic pollutants (POPs) (i.e., polybrominated diphenyl ethers (PBDEs), organochlorine pesticides, and polychlorinated biphenyls (PCBs)) in dust collected from pregnant African Americans (AAs) in Atlanta using isotope dilution gas chromatography-tandem mass spectrometry. Limits of quantification were ranged from 0.10 to 2.50 ng/g dust. NIST standard reference material measurements demonstrated the robustness of our method. Our accuracies ranged from 82 to 108%, relative standard deviations ranged from 2 to 16%, and extraction recoveries ranged from 76 to 102%. We measured POPs in dust collected from 34 homes of pregnant AAs participating in the Atlanta AA birth cohort study who were enrolled from 2016 to 2019. Concentrations of POPs were detected in all samples with the frequencies of detection ranging from 8 to 100%. Concentrations of PBDE congeners 99 and 47, *p*,*p*’-DDT, and PCB153 were detected at some of the highest concentrations with geometric means of 1270, 730, 63.4 and 240 ng/g, respectively. The ratio of DDT/DDE was quite large (~2.7) indicating that *p*,*p*’-DDT remains intact in homes for long periods of time. These data demonstrate that care should be taken to remediate POPs in indoor dust, especially in vulnerable, disparate segments of the population.

## 1. Introduction

**Figure 1:**
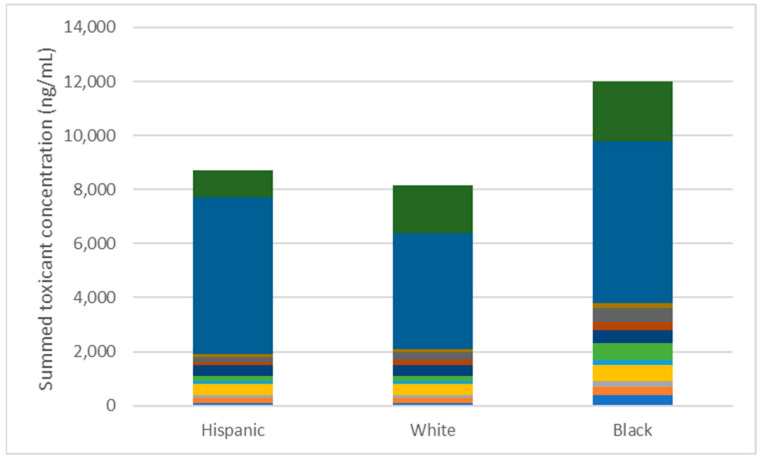
Summed body burden of 59 chemicals in the National Health and Nutrition Examination Survey 2008–2009 dataset stratified by race/ethnicity demonstrate that non-Hispanic Blacks have higher total chemical burden. About 80% of the chemicals evaluated were higher in non-Hispanic Blacks than other racial/ethnic groups.

Over 80,000 different chemicals have been introduced into manufacturing with few of these having appropriate toxicity or exposure testing prior to their use [1]. As a result, chemical contamination of the environment is pervasive [2,3,4,5,6,7,8]. Beginning in 1999, the Centers for Disease Control and Prevention’s National Health and Nutrition Examination Survey (NHANES) began measuring “background” levels of ~300 environmental chemicals in humans demonstrating widespread human exposures with distinct race, age, and sex disparities [9,10,11,12,13,14,15,16,17]. Non-Hispanic Blacks (NHB) had higher levels of approximately 80% of the environmental toxicants measured than did Mexican Americans or non-Hispanic whites [18]. More specifically, levels of bisphenol A (BPA), an estrogenic and pervasive environmental contaminant, plasticizer phthalates, markers of air pollution such as polycyclic aromatic hydrocarbons (PAHs) and cotinine, antimicrobial parabens, brominated flame retardants, and pesticides were all higher in NHB than in other racial/ethnic groups [18] (Figure 1). Total toxicant burden in NHB was 30–50% higher than other demographic groups [18]. Clearly, the higher and disparate exposures that African Americans (AAs) experience in the US population puts them at greater risk of developing environmentally-related disease, in addition to the disadvantages they experience from structural racism and environmental injustice [19,20,21,22]. Despite the racial inequities apparent in chemical exposures in the United States, few studies have focused on population-based exposures in AAs in the Southeast despite regional differences in climate, housing, population density, culture, and racial/ethnic composition [23,24,25,26,27].

A potentially large contributing pathway to this exposure is harmful chemicals present in house dust [7,28,29,30,31]. Many common environmental chemicals, including metals, pesticides, phthalates, and polycyclic aromatic hydrocarbons, sequester in house dust [28,32]. These dust particles, around 5–25 µm in size, resuspend in the air from home activities such as walking, vacuuming, and sweeping [33]. Adults, on average, ingest 50 mg of dust and inhale 0.8 mg daily day [32]. Due to different activity patterns and breathing rates, children ingest ~100 mg and inhale 2 mg dust per day [32]. House dust particles can remain in the home for decades as the particles continuously resuspend in the air, collect on surfaces, and resuspend again [28,32]. Therefore, house dust provides a near constant exposure to the various harmful chemicals that sequester in this medium.

Persistent Organic Pollutants (POPs), such as polybrominated biphenyls (PCBs), polybrominated diphenyl ethers (PBDEs), dioxins, and organochlorine pesticides, are some of the harmful compounds that can be found in house dust [28,29,31,32]. POPs are resistant to biodegradation, so they remain in the environment for long periods of time [34,35,36]. Although many POPs were banned as a part of the Stockholm Convention, their slow environmental degradation and high bioaccumulation and biomagnification means that most all people are still exposed to them regularly [16,17]. POPs have multiple adverse environmental and human health impacts [4,5,34,37,38,39,40,41,42]. In wildlife, exposure to POPs causes thinning of eggshells, skeletal deformations, and reproductive failure [37,38,39]. Artic wildlife has experienced severe effects including immunotoxicity and disruption of development due to the elevated levels of POPs that gather in the artic regions [6,38,39]. In humans, chronic exposure to POPs has been linked to cancer, neurodevelopmental deficits, adverse reproductive outcomes, and other adverse effects [37,42,43,44]. Furthermore, pregnant women’s exposure to POPs has been correlated with complications in gestation and post-partum in mothers and low birth weight and fetal deaths in infants [23,25,42,45,46,47,48,49].

Therefore, to understand POPs exposure from dust in the Southeast better, we aimed to measure multiple POPs in the house dust of AA families’ homes in Atlanta, Georgia to quantify exposures experienced by this underrepresented group. We selected the specific PCBs and PBDEs measured in this study because of their biological relevance. The PCBs we measured represent about half of the total body burden of PCBs and are the ones used to calculate summed PCBs [17,50].

## 2. Methods

### 2.1. Chemicals and Reagents

Analytical grade dichloromethane (DCM), n-hexane, and nonane were purchased from Sigma-Aldrich, Inc. (St. Louis, MO, USA). Ethyl acetate and methanol were purchased from Fisher Scientific (Waltham, MA, USA). Water was generated using a Milli-Q Ultrapure water purification system (Millipore, Billerica, MA, USA). The standard reference material (SRM 2585, organic contaminants in house dust) was purchased from the National Institute of Standards and Technology (NIST, Gaithersburg, MD, USA). Helium and nitrogen gases were of 99.999% ultra-high purity and obtained from nexAir, Inc. (Suwanee, GA, USA). Florisil solid phase extraction (SPE) columns (200 mg/6 mL) were purchased from Phenomenex (Torrance, CA, USA).

Individual PCB congeners (PCB 118, 153, 138 and 180) at 100 µg/mL in isooctane, individual PBDE congeners (PBDE 47, 85, 99, 100, 153, and 154 at 50 ug/mL in nonane, and organochlorine pesticides [*p*,*p*’-dichlorodiphenyltrichloroethane (*p*,*p*’-DDT), *p*,*p*’-dichlorodiphenyldichloroethylene (*p*,*p*’-DDE), and trans-nonachlor (TNC)] at 100 µg/mL in nonane were purchased from Cambridge Isotope Laboratories (Andover, MA, USA). The purity of all native standards was ≥95%.

A mixed PCB congener (5 µg/mL) solution in nonane, a mixed PBDE congener (7.50 ng/mL) in methanol, and individual standards for *p*,*p*’-DDT, *p*,*p*’-DDE, and TNC at 100 µg/mL in nonane, all ^13^C-ring labeled, were purchased from Cambridge Isotope Laboratories. The stable isotope labelled standards had >99% isotopic purity.

A set of 10 levels PBDE calibration solutions (CS1–CS10), ranging from 0.20 to 2000 ng/mL native analyte, was purchased from Cambridge Isotope Laboratories (Andover, MA, USA). The calibrants also contained ^13^C_12_ labeled analyte at 75.0 ng/mL.

### 2.2. Preparation of Standard Calibrants and Labeled Standard Spiking Solutions

Two sets of calibration standards were used to quantify all target analytes. All PBDE congeners were quantified using a set of calibration standards (CS1–CS10) mentioned above. The calibration curve was prepared by diluting each CS standard calibrant with nonane 4 times to yield a calibration range of 0.05–500 pg/uL. The PBDE calibrant concentrations were expressed in dust equivalents as 0.10–1000 ng/g dust. The other analytes were quantified using a set of calibration standards made in house. The calibration standards were made by mixing all native analytes (non-PBDEs) and serially diluted with nonane to yield a calibration range of 0.40–1000 pg/uL. The calibrant concentrations were expressed in dust equivalents as 0.40–1000 ng/g dust. All calibrants were solvent-based standards made in nonane.

For analytes other than the PBDE congeners, a mixture of labeled standard solution was made in methanol to yield a concentration of 100 ng/mL for PCB congeners and 200 ng/mL for *p*,*p*’-DDT, *p*,*p*’-DDE, and TNC. All standard solutions and spiking solutions were prepared or dispensed into amber vials and stored at 4 °C until used.

### 2.3. Quality Control and Assurance

A dust pool was made by mixing residual dust samples together in a polypropylene tube with a screw cap and were mixed vigorously to homogenize. From this dust pool, four dust samples were aliquoted, prepared, and analyzed alongside the unknown samples to evaluate method precision. Duplicates of reagent blank samples were prepared and analyzed alongside the unknown samples to evaluate potential contamination. Four samples of the NIST SRM 2585, organic contaminants in house dust, were also prepared and analyzed to ensure the accuracy of the data.

### 2.4. Study Design and Population

House dust samples (*n* = 34) were collected from the homes of participants of the Atlanta AA Maternal-Child Cohort (ATL AA hereafter) around gestational week 20 and again after the child’s birth. This ongoing, prospective birth cohort enrolls pregnant AAs between 6 and 17 weeks gestation at Emory Midtown Hospital and Grady Hospital, which serve socioeconomically diverse populations in Atlanta, Georgia, and extends dyad follow-up through age five. Additional information regarding the cohort profile and data collection is described in detail elsewhere [23,24]. Participants were eligible for inclusion if they self-identified as AA, born in the US, between 18 and 40 years old, pregnant with a singleton pregnancy, fluent in English, and had no chronic medical conditions [51]. All participants provided written, informed consent to participate in the study, which was approved by the Institutional Review Board at Emory University (approval reference number 68441). Questionnaire data collected included potential sources of chemical exposures such as furniture and housing age, water source, smoking status, cleaning product use, and other common chemical exposure sources.

### 2.5. Dust Collection

For dust sample collection, a 1 m^2^ grid was placed at a high traffic area in the home, preferably carpeted. Dust was obtained by vacuuming the area inside the grid in all 4 directions to maximize uptake using a standard household vacuum cleaner (Hoover, Charlotte, NC, USA) and X-100 dust sock attachments (Midwest Filtration LLC, Cincinnati, OH, USA). Dust socks are 9 × 4 in mesh collection socks with a particle retention efficiency of 97% (≥1 µm) which is mounted on a one-use, disposable cardboard mounting and crevice tool that enables the collection of dust samples without contamination from the vacuum source. The dust was emptied from the dust sock into an amber 2 oz Qorpak bottle (Clinton, PA, USA) until processed at the Laboratory for Exposure Analysis and Development in Environmental Research (LEADER). In the laboratory, dust samples were course sieved to 250 µm using a 8(round) × 2 in, brass No. 60 Mesh Testing Sieve (ThermoFisher Scientific, Waltham, MA, USA) followed by a fine sieve to 150 µ using a similar sieve with No 100 Mesh. The resulting finely sieved dust was weighed and stored in an amber 1 oz Qorpak bottle (Clinton, PA, USA) until analyzed.

### 2.6. Sample Preparation and Analysis

For analysis, 50 mg of sieved dust was weighed into a sample tube, spiked with isotopically labeled analogues of the target chemicals (125 µL for PBDE labeled solution and 50 µL for other labeled mixture), and mixed with 2.5 mL DCM. The mixture was vortex mixed for 2 min followed by a 5 min sonication. The mixture was centrifuged for 10 min (1000× *g*). The supernatant was allowed to pass through a Strata Florisil solid phase extraction (SPE) column (200 mg/6 mL) using gravity while collecting the eluate. Liquid extraction using DCM was done again and the supernatant was loaded onto the same Florisil SPE cartridge. The Florisil SPE cartridge was then eluted first with 8 mL of n-hexane and then 10 mL of ethyl acetate. The eluate was evaporated to dryness using a TurboVap^®^ (Zymark, Framingham, MA, USA) set at 50 °C and 15 psi. The extracts were reconstituted in 50 µL nonane prior to injection. Calibrants, blanks, and NIST standard reference were analyzed concurrently with unknown dust samples. Samples were analyzed using gas chromatography-tandem mass spectrometry (GC-MS/MS) using an Agilent 7890 A GC coupled to an Agilent 7000B triple quadrupole mass spectrometer (Agilent Technologies, Santa Clara, CA, USA) with electron impact (EI) ionization. The system was programmed and controlled using MassHunter Workstation Software version B.05.00. Calibration and tuning of the instrument were performed in the EI mode with High Sensitivity Autotune mode, and instrumental performance was always checked prior to analysis.

The GC system was fitted with a Zebron ZB-5 (5%-phenyl-95%-dimethylpolysiloxane) analytical column (30 m × 0.250 ID × 0.25 μm film thickness, Phenomenex, Torrance, CA) for optimum separation. A 2 μL injection was used with an injection port temperature set to 280 °C in pulsed splitless mode. The helium carrier gas flow rate was 1 mL/minute through the end of the run, with a quench helium and collision N_2_ gas flow rates of 2.25 mL/minute 1.25 mL/minute, respectively. The oven temperature program was as follows: 90 °C (0.5 min), ramped to 250 °C (40 °C/min), ramped to 270 °C (5 °C/min), and held for 5.5 min. The total run time was 16 min. Transfer line, source, and quadrupole temperatures were set to 280 °C, 230 °C, and 150 °C, respectively. Two transitions were monitored for each native analyte for quantification and confirmation. Only one transition was selected for each labeled analyte. All transitions were monitored in a multi-segment analysis using multiple reaction monitoring (MRM) mode, with unit resolution for MS1 and wide resolution for MS2. These MRM transitions and associated parameters are summarized in Table 1.

To quantify values, calibration plots were derived from the linear or quadratic regression analysis of the standard concentrations (in dust equivalents) plotted against the area of the native standard/area of the labeled standard. Sample analyses were only considered valid if the analyte was present at the same retention time as its labeled analogue and had agreement among concentrations calculated from both the quantification and confirmation ions. In addition, NIST materials had to fall within 20% of the certified values or the entire sample run was repeated. We evaluated the utility of our method to calculate POP levels in house dust by analyzing these toxicants in samples collected from our ATL AA cohort homes.

### 2.7. Validation

The limits of quantification (LOQs) were set based on the lowest calibrant in the calibration curve that meets the following criteria: the accuracy of 80–120%, the precision of ±20%, and calculated concentration was ≥5 times than that of blank samples. Accuracies were determined by noting the agreement of our calculated values of NIST SRM samples to the NIST certified values. Relative standard deviations (RSDs) were from repeated measured of pooled dust samples.

### 2.8. Statistical Analysis

The data were processed and statistically analyzed using Microsoft Excel 2017 (Microsoft Corporation, Redmond, WA, USA) and SAS Statistical Software Version 9.4 (SAS Corporation, Cary, NC, USA). Prior to descriptive data analysis of the target analyte concentrations, the concentrations below the LOQ were assigned a value equal to the LOQ/√2 [52]. Geometric means (GMs) and various distribution quantiles were calculated along with the frequencies of detection.

## 3. Results

Six retention windows were specified in our data acquisition program to maximize sensitivity. Each run was 15 min long allowing the analysis of up to 96 samples per day. However, our rate limiting step was in sample preparation which allowed 28 unknowns to be easily processed by one lab analyst per day followed by 10.5 h of GC-MS/MS run time.

Specifications of our methods are shown in Table 2. Extraction recoveries ranged from 76 to 102% which were sufficient to provide low LOQs appropriate for dust measurements. Our LOQs were in a range of 0.10 ng/g dust to 2.50 ng/g dust. PBDE 47 has the lowest LOQ value (0.10 ng/g dust). PBDE 153 and PBDE 154 have the highest LOQ value (2.50 ng/g dust) likely due to peak broadening and heat-induced debromination in the GC injection port, a phenomenon that has been previously reported [53].

The measurement accuracies based upon NIST SRM 2585 ranged from 81.7 to 107.5%. Relative standard deviations determined from repeated measurements of pooled dust samples and NIST materials were below 15%. According to FDA analytical method guidelines, accuracies ranging from 80 to 120% and RSDs ≤ 15% are considered acceptable for proper analytic measurements [54].

**Figure 2:**
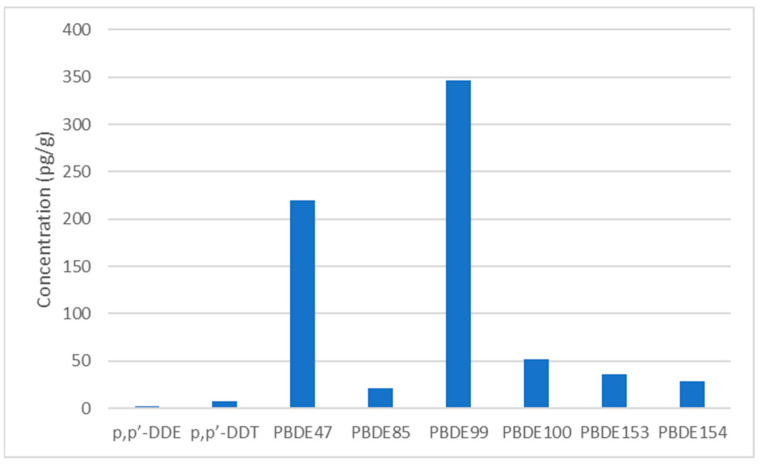
Median concentrations of the most prevalent persistent organic pollutants detected in Atlanta house dust, 2016–2019.

The distribution of POPs measured in dust samples is provided in Table 3 and Figure 2. PBDE congeners measured in this method were detected in 82–100% of the dust samples with PBDE 99 having the highest concentration with an GM of 1267 ng/g. This is consistent with the relative abundance of each PBDEs in our NIST SRM material. The organochlorine pesticides TNC, *p*,*p*’-DDE and *p*,*p*’-DDT were detected in 85–100% of the dust samples tested; *p*,*p*’-DDT was measured at the highest concentration with a GM of 63 ng/g. PCBs were detected more sporadically with PCB138 being the most frequently detected and PCB180 being detected infrequently (8%).

If we assume that the average adult intake occurs at the GM level, the total consumption of PBDEs via inhalation for a 70 kg adult would be equivalent to 109 ng/day resulting in a dose of 1.6 ng/kg/day. Similarly for a 14 kg child (~3-year-old child), intake of PBDEs per day would be 223 ng/day (range <LOQ to 2361 ng/day) resulting in a dose of 16 ng/kg/day (range <LOQ to 169 ng/kg/day). For DDT, adult intake would be 280 pg/day or 4.0 pg/kg/day (range <LOQ to 628 pg/kg/day) and for a child, it would be 570 pg/day or 41 pg/kg/day (range <LOQ to 75 pg/kg/day).

## 4. Discussion

One interesting observation in our study was the unexpected ratio of DDT/DDE in house dust in Atlanta. Typically, we would find *p*,*p*’-DDE at higher levels because of the environmental weathering effect on *p*,*p*’-DDT. Given that most of the DDT to DDE conversion in the environment is microbial [55] with a soil half-life of 2–15 years [56], we would not expect the ratio to be comparable necessarily to soil levels (~1/10) unless the indoor dust levels resulted from tracking soil into the home, but we did expect the *p*,*p*’-DDE levels to be higher than its parent [57]. On average, *p*,*p*’-DDT levels were three times higher than the *p*,*p*’-DDE levels in our study. This ratio is consistent with the ratio recently reported in rural Nepal, but the authors surmise that their observation resulted from a fresh application and minimal degradation of DDT in their local environment. Given that *p*,*p*’-DDT registrations were all eliminated in the United States in 1972 [58] and assuming no illegal applications occurred in our study population homes, *p*,*p*’-DDT would have remained present and detectable in the house dust for over five decades despite home cleaning and vacuuming performed to reduce dust levels.

To contextualize our findings, we compared our data to those reported in other studies (Table 4). Clearly, great spatial and temporal variability in dust POP levels exists even within a geographic region like North America. Most studies have focused on exposures in California which has strict chemical use regulations that can impact dust POP levels or along the eastern seaboard. To date, only one other study has reported levels of POPs in house dust in the Deep South and it focused only on PBDE levels. Our PBDE congener levels were higher than most others reported, except for California which has historically higher levels than other US states because of its past use to meet strict fire safe-guarding regulations [59]. The levels of PBDE 47, one of the predominantly detected PBDE congeners in dust, was almost double the levels evaluated in racially diverse children’s homes in Atlanta during a similar timeframe. Surprisingly though, although PBDE 47 is found at the highest concentrations in serum samples, it was not found at the highest levels in our house dust. PBDE 99, a congener resulting from the use of the same Penta mixture that PBDE 47 derives from, was detected at the highest concentrations. This is the opposite of what we find in our participants serum for which the median of PBDE 47 was almost three times higher than the PBDE 99 levels. Our findings suggest high exposures to POPs are occurring in our population of pregnant women in Atlanta.

Our study has several strengths and limitations. Our sample size is small and contains data on a select number of POPs. We eventually plan to collect more samples from our cohort and expand the number of chemicals that we can easily measure in dust to include other POPs and non-persistent toxicants. Ideally, we will ultimately be able to measure the full complement of chemicals that are currently measured in the US NHANES study. Furthermore, these data could be used in a more focused risk assessment of our population enabling us to better understand the real health risk posed by this exposure pathway. However, these data do represent the first in the Atlanta area, particularly in a vulnerable and underserved segment of our population, that demonstrate a potentially modifiable pathway of exposure to toxic chemicals. Given these results, further study is warranted.

## 5. Conclusions

Our study is the first to characterize house dust concentrations in the homes of an AA population in the Deep South/Southeast. These data suggest that our extraction and acquisition methods are sensitive enough to detect and quantify concentrations of multiple organic pollutants found in house dust. The concentrations of POPs in dust ranged from <LOD to almost 25,000 ng/g dust, suggesting that dust may be an important pathway for POP exposure in our population. PBDEs and organochlorine insecticides were detected most frequently in more than 85% of the samples tested with PCBs being detected less frequently and at lower concentrations.

## Figures and Tables

**Table 1:** MRM transitions and related parameters by target compound.

Compound	Ion Type	Precursor Ion (*m*/*z*)	Product Ion (*m*/*z*)	Dwell Time (ms)	Collision Energy (V)	Retention Window	Approx. RT (min)
TNC	Q	406.7	299.8	75	25	1	
TNC	C	408.6	299.9	75	25	1	6.79
*p*,*p*’-DDE	L	257.9	188.2	75	35	1	
*p*,*p*’-DDE	Q	245.9	176	75	35	1	6.87
*p*,*p*’-DDE	C	247.9	176	75	35	1	
PCB 118	Q	323.7	254	50	20	2	
PCB 118	C	325.6	256	50	40	2	7.30
*p*,*p*’-DDT	L	247	177.2	50	25	2	7.87
*p*,*p*’-DDT	Q	234.9	165	50	25	2	
*p*,*p*’-DDT	C	236.9	165	50	25	3	
PCB 153	L	373.7	301.9	50	30	2	
PCB 153	Q	359.7	289.9	50	40	2	
PCB 153	C	361.6	289.9	50	40	2	7.57
PCB 138	L	373.7	301.9	50	30	3	
PCB 138	Q	359.7	289.9	50	40	3	
PCB 138	C	361.6	289.9	50	40	3	7.96
PCB 180	L	405.8	335.7	75	30	4	
PCB 180	Q	393.6	324	75	30	4	
PCB 180	C	395.6	323.8	75	30	4	9.01
PBDE 47	L	497.8	338	75	30	4	
PBDE 47	Q	485.6	326	75	30	4	
PBDE 47	C	325.7	217	75	30	4	9.10
PBDE 85	Q	565.5	406	75	20	6	s
PBDE 85	C	563.6	404	75	20	6	13.48
PBDE 99	L	577.7	417.7	75	25	5	
PBDE 99	Q	563.5	404	75	25	5	
PBDE 99	C	565.5	406	75	25	5	11.80
PBDE 100	L	577.7	418	75	25	5	
PBDE 100	Q	563.5	404	75	25	5	
PBDE 100	C	565.5	406	75	25	5	11.09
PBDE 153	L	655.5	495.5	100	20	7	
PBDE 153	Q	643.5	484	100	20	7	
PBDE 153	C	483.5	324	100	20	7	14.98
PBDE 154	L	655.5	495.5	100	20	6	
PBDE 154	Q	643.5	484	100	20	6	
PBDE 154	C	641.5	482	100	40	6	14.36

Q = quantification ion; C = confirmation ion; L = labeled isotope ion; MRM = multiple reaction monitoring; RT = retention time; *p*,*p*’-DDE = dichlorodiphenyldichloroethylene; *p*,*p*’-DDT = dichlorodiphenyltrichloroethane; PCB = polychlorinated biphenyl; PBDE = polybrominated biphenyls; TNC = trans-nonachlor; CE = collision energy.

**Table 2:** Validation specifications of the method.

Analyte	LOQ (ng/g Dust)	Accuracy (%) *	Recovery (%) **	RSD (%)	
				Low QC	High QC
TNC	2.00	81.8	88 ± 6	15.6	3.0
*p*,*p*’-DDE	0.40	92.2	92 ± 4	10.2	1.3
*p*,*p*’-DDT	1.00	96.8	88 ± 10	9.4	3.3
PCB118	1.00	85.9	91 ± 5	11.2	5.6
PCB138	1.00	92.9	86 ± 11	9.9	4.7
PCB153	1.00	96.7	101 ± 6	6.1	3.4
PCB180	2.00	100.2	93 ± 4	12.0	2.6
PBDE47	0.10	89.2	102 ± 4	10.2	5.9
PBDE85	0.25	107.6	81 ± 11	11.6	10.2
PBDE99	0.50	94.5	97 ± 5	9.5	6.7
PBDE100	2.50	97.4	95 ± 7	9.1	4.8
PBDE153	2.50	90.5	98 ± 5	13.1	11.9
PBDE154	2.50	84.8	105 ± 9	12.2	10.7

* = based upon NIST Standard reference material 2585; ** = extraction recovery; QC = quality control material; RSD = relative standard deviation; FOD = frequency of detection; LOD = limit of detection; *p*,*p*’-DDE = dichlorodiphenyldichloroethylene; *p*,*p*’-DDT = dichlorodiphenyltrichloroethane; PCB = polychlorinated biphenyl; PBDE = polybrominated biphenyls; TNC = trans-nonachlor.

**Table 3:** Distribution of persistent organic pollutants in dust samples, Atlanta, 2016–2019 (ng/g).

Analyte	FOD (%)	GM	Median	95th%ile	Maximum
TNC	85.3	4.90	6.54	71.5	356
*p*,*p*’-DDE	100	3.45	2.46	57.6	356
*p*,*p*’-DDT	85.3	5.59	7.08	227	880
PCB118	23.5	<1	<1	3.07	11.2
PCB138	41.2	<1	<1	3.04	95
PCB153	17.6	8.8	85.3	7.84	239
PCB180	8.8	<0.1	<0.1	1.98	4.62
ΣPCB_4_	44	3.59	<2.17	15.6	246
PBDE47	100	252	220	3078	6741
PBDE85	100	24.4	20.8	465	519
PBDE99	100	380	347	6779	8167
PBDE100	100	64.5	51.9	1216	1720
PBDE153	100	44.5	35.8	774	888
PBDE154	100	34.2	28.5	603	801
ΣPBDE_6_	100	2187	1628	18,400	23,150

*p*,*p*’-DDE = dichlorodiphenyldichloroethylene; *p*,*p*’-DDT = dichlorodiphenyltrichloroethane; PCB = polychlorinated biphenyl; PBDE = polybrominated biphenyls; TNC = trans-nonachlor; ΣPCB_4_ = sum of all 4 PCB congeners; ΣPBDE_6_ = sum of all 6 PBDE congeners.

**Table 4:** Dust POP concentrations in various studies.

Citation	Year	Location	N	Median/GM PCB 153 ng/g	Median/GM *p,p*-DDE ng/g	Median/GMPBDE 47 ng/g
This study	2016–2020	Atlanta, GA, USA	34	240	18	720
Darrow et al., 2017 [29]	2011–2012	Atlanta, GA, USA	15	NQ	NQ	404
Whitehead et al., 2013, 2014, 2015 [36,59,60]	2001–2007, 2010	CA	292, 203	NQ	NQ	1500, 1300
Johnson et al., 2010 [61]	2002–2008	MA	50	NQ	NQ	390
Tan et al., 2007 [62]	NP	Singapore	31	0.5 (ΣPCB)	3.3	NQ
Meng et al., 2016 [63]	2014	Shanghai, China	60	Case: 0.09 Control: 0.08	Case: 32 Control: 16.36	Case: 3.02 Control: 1
Chandra Yadav et al., 2019 [64]	NP	Nepal	200	1–2.89 (ΣPCB)	91–371 (∑DDT)	NQ
Shoeib et al., 2012 [65]	2007–2008	Vancouver, Canada	116	NQ	NQ	280

GM = geometric mean; N = number of samples tested; NQ = not quantified, ND = not detected, NP = not provided.

## Data Availability

The data presented in this study are available on request from the corresponding author.
